# The role of soft skills and multicultural attitudes in enhancing teaching quality in Colombian higher education

**DOI:** 10.1371/journal.pone.0321490

**Published:** 2025-04-11

**Authors:** Fernando Barrios Aguirre, Susan Benavides Trujillo, Edgar Iván Vega Guillén, Fernando Alonso Téllez Mendivelso

**Affiliations:** 1 Escuela de Negocios, Fundación Universitaria Konrad Lorenz, Bogotá, Colombia; 2 Vice-Rector for Research and Outreach, Universidad de América, Bogotá, Colombia; 3 National Deputy Director for Curriculum, Fundación Universitaria del Área Andina, Bogotá, Colombia; 4 Vice President for Academic Affairs, Universidad del Istmo, Panamá, Panamá; Faculty of Electrical Engineering, Computer Science and Information Technology Osijek, Croatia

## Abstract

This paper will explore whether teachers’ multicultural ideology, soft skills (transversal capacities), national pride and identity, and intergroup contact, controlled by other variables, are related to their multicultural attitudes. This objective is developed and estimated from correlation analysis and using the method of ordinary least squares, to verify how multicultural ideology, national pride and identity, soft skills, and intergroup contact, controlled for some sociodemographic variables, generate effects on the multicultural attitudes of teaching in a Colombian higher education institution with high-quality accreditation. The findings revealed that soft skills and multicultural ideology positively influenced teachers’ multicultural attitudes, while intergroup contact with different ethnic groups showed a negative correlation. The analysis used primary data collected from 199 professors at the institution, which operates in a culturally diverse, multi-campus context within an emerging country characterized by very low national pride.

## Introduction

The complexities and contradictions of today’s world have created new challenges, generating tensions for which education must prepare individuals. Education must enable people to adapt and respond effectively to these changes. Consequently, it has become necessary to rethink education and learning within this evolving context.

As a global observatory of social transformations, UNESCO has prioritized fostering public debates on educational policies. Among these debates is the discussion of the skills students need to adapt to this new society. Higher education plays a role that extends beyond preparing students for future employment. It also involves equipping future professionals with both soft and hard skills, ideally fostering innovation and enabling them to address global and local (“glocal”) problems. Additionally, higher education institutions must promote innovation and meet the demands for effective and efficient training systems. While education plays a central role in developing innovation skills, various studies suggest that higher education institutions have struggled to meet these demands. Therefore, updating pedagogical practices and developing evaluation tools to measure and enhance individuals’ innovation capacity has become essential [1].

Critics have argued that educational practices, particularly in higher education, often fail to adequately develop the skills necessary for innovation [2,3,4,5]. Previous studies have shown that the skills required for innovation activities are not effectively integrated into teaching or academic assessment. As a result, updating higher education curricula and redesigning assessment structures has become imperative [6,7].

Vila et al. [4] emphasized the importance of students acquiring specific skills and competencies during their studies to participate in innovative activities in the workplace. According to Bath, Smith, Stein, and Swann [8], these skills develop most effectively when integrated as learning objectives in study plans. Learning outcomes describe what students are expected to know, understand, and demonstrate by the end of a learning period. These statements outline the achievements and competencies assessed at the end of a course. This approach requires a shift from a testing culture to one that supports learning, promotes student-centered and active agency, and values process assessment over end products. In essence, holistic assessments should incorporate self-assessments and peer assessments, which play a critical role in future learning in higher education [9].

Soft skills encompass the abilities and competencies necessary for personal, group, and societal interactions [10,11]. Individuals use these skills to communicate their ideas and perceptions effectively. Intrapersonal skills, on the other hand, refer to self-management abilities. When individuals possess strong interpersonal skills, others perceive them as more competent, which boosts their self-confidence and sense of value [12].

Updating teacher training programs and orienting them toward preparing globally oriented teachers has become increasingly important [13]. However, the rapidly changing world has created complex and often confusing circumstances for teacher education colleges [14]. Among the most prominent challenges are curriculum overload, conflicts between local politics and global attitudes, and vague or misguided ideas about certain topics or individuals.

Attitudes toward multiculturalism in educational contexts play a crucial role in creating inclusive and respectful learning environments. These attitudes are desirable qualities for effective educational practices among both teachers and students. However, little is known about the background of teachers’ and students’ multicultural attitudes. Factors such as multicultural ideology, national pride and identity, and intergroup contact influence these attitudes. Assessing multicultural attitudes (using an adapted version of the Teachers’ Multicultural Attitude Survey), multicultural ideology (using the Multicultural Ideology Scale), national pride and identity (a unique element of large-scale studies), and intergroup contact (experiences in multicultural environments, classrooms, and intergroup friendships) provides valuable insights.

Attitudes toward multiculturalism in educational contexts vary depending on factors such as cultural environments, the openness of the social environment to multicultural perspectives, the exposure of students and teachers to diverse settings, their prior education, educational policies, and the social history of the country in question [14]. In the context of education, multiculturalism serves as an important tool for promoting respect, tolerance, and understanding among individuals from different cultures and ethnic groups. However, some individuals may hold negative attitudes toward multiculturalism due to a lack of information or awareness about other cultures, fear of the unknown, or the belief that cultural diversity threatens social cohesion or national identity.

This manuscript examines the principal determinants of teachers’ attitudes toward multiculturalism in educational contexts to better understand this phenomenon and its impact on education. Higher education institutions in Colombia should consider implementing policies and programs that support diversity and inclusivity, as well as training and professional development programs that enhance soft skills. This case study includes primary data from 199 professors at a Colombian university with institutional accreditation, operating in a culturally diverse context within an emerging country with low national pride. Previous studies have emphasized the importance of fostering innovation and adaptability in students by integrating soft skills into curricula [1,15]. However, research on how these competencies influence teachers’ multicultural attitudes remains scarce, particularly in emerging economies. Most studies focus on developed nations, often overlooking the unique socio-cultural and economic factors affecting multicultural education in regions like Colombia [16]. This study addresses this gap by analyzing the role of multicultural ideology, soft skills, national pride, and intergroup contact in shaping teachers’ multicultural attitudes. By examining these variables in a Colombian higher education institution with institutional accreditation, the study provides fresh insights into the interaction between multicultural perspectives and pedagogical practices. This research contributes to the discourse on internationalization in higher education by highlighting specific challenges and opportunities in multicultural teaching environments.

The findings suggest that attitudes toward multiculturalism in educational contexts are desirable qualities for effective teaching practices and for understanding student learning dynamics. The results indicate a positive effect of soft skills, national pride, science diplomacy, and multicultural ideology on teachers’ multicultural attitudes, and a negative effect of knowledge of friends and peers in other ethnic groups on these attitudes. The paper concludes with a discussion of the implications for higher education institutions in Colombia, emphasizing the need to update educational practices to address multiculturalism and foster the development of soft skills in higher education. The study also underscores the significance of teachers’ multicultural attitudes and their impact on teaching effectiveness and student learning dynamics in Colombia, a context that remains underexplored.

This article is divided into five sections. The first section presents the literature review, focusing on how multicultural ideology, soft skills, national pride and identity, and intergroup contact relate to multicultural attitudes, while controlling for other variables. The second section outlines the econometric model, data, and identification strategy for the estimated effects in the ordinary least squares (OLS) model. The third section presents descriptive statistics and analyzes the research results. Finally, the fourth and fifth sections discuss the findings and present the conclusion.

## Literature review

Teachers with well-developed soft skills demonstrated more positive multicultural attitudes, fostering a deeper understanding, appreciation, and valuation of cultural diversity. Those who embraced diversity were generally more effective in multicultural contexts, which enhanced their ability to teach students from diverse backgrounds. Consequently, integrating multicultural education into teacher training programs became essential for equipping future educators with the competencies required for culturally diverse classrooms.

The transmission of multicultural education by teachers played a critical role in preparing students to develop competencies associated with culturally diverse environments. For this reason, experts argued that these aspects should become essential components of curricula, the internationalization of professions, and intercultural teaching methods. These elements promoted and appreciated a culturally diverse world. Educational institutions at the secondary and university levels held a crucial role in fostering these competencies. They provided students with the necessary skills and knowledge to navigate an increasingly globalized labor market and adapted curricula to align with technological advancements and evolving professional demands. Understanding multiculturalism and its integration into education enabled students, teachers, and administrators to create welcoming learning environments that promoted problem-solving, cross-cultural understanding, and sustainable development.

Recognizing the complexity of multiculturalism and the benefits of incorporating multicultural education in higher education institutions led students, teachers, and administrators to create inclusive environments that improved the learning process. Education systems worldwide needed to foster global competencies, including problem-solving, understanding different perspectives, positive interactions, and actions toward sustainable development and collective well-being.

Teacher training programs required updates to equip educators with the skills and knowledge needed to teach effectively in multicultural and globally interconnected environments. However, these programs often faced challenges such as curriculum overload and tensions between local political priorities and global perspectives. Addressing these challenges demanded the identification and implementation of clear strategies that integrated essential multicultural knowledge, skills, and values into teacher education. Prior research suggested that soft skills, national pride, and multicultural ideology positively influenced teachers’ multicultural attitudes, while knowledge of friends and peers from other ethnic groups sometimes had a negative impact. Thus, initial teacher education programs needed to emphasize the reinforcement of multicultural ideology while also considering the roles of national pride and soft skills.

Existing literature inspired this research by highlighting the positive effects of soft skills, national pride, and multicultural ideology on teachers’ multicultural attitudes, as well as the potential negative effects of knowledge of friends and peers from other ethnic groups. For this reason, the objectives of this research focused on how initial teacher education programs could reinforce and develop the multicultural ideology of future teachers while considering the roles of national pride and soft skills. The literature also emphasized three key aspects: the internationalization of education and science diplomacy, the multicultural attitudes of actors, and the relationship between soft skills, multiculturalism, and science diplomacy.

Cultural diversity emerged as a determining factor in attitudes toward multiculturalism in educational contexts. Schools with diverse and multicultural student bodies often fostered more positive attitudes toward multiculturalism. Additionally, knowledge and understanding of other cultures through education increased acceptance and respect for different cultures. When teachers and students understood the global world through the multicultural experiences they acquired throughout life, they could more easily transmit their multicultural attitudes to any context, including education. Respect, human understanding, interactions, and cultural and ethical understanding primarily stemmed from these experiences. In this sense, positive reminders of multiculturalism led to more positive interactions with people from many cultures, while negative reminders generated belligerent attitudes toward other cultures.

Intercultural education created an environment of preparation and assimilation of positive attitudes toward multiculturalism before direct experiences occurred. To prepare educators for multicultural environments, curricula needed to include intercultural content that encouraged the understanding and appreciation of different cultures while promoting tolerance and acceptance. Social and cultural influences throughout life, including interactions with parents, friends, and the broader community, significantly impacted individuals’ receptiveness to multiculturalism. Moreover, educational policies that supported cultural diversity and intercultural education helped foster positive multicultural attitudes.

Finally, the development of positive views toward multiculturalism benefited from educational policies that supported cultural variety and intercultural education and created opportunities for human interaction. Teachers played an important role in the development of students’ learning and in their motivation to achieve academic, professional, personal, and social success. Soft skills, defined as a set of intrapersonal and interpersonal skills related to behaviors and attitudes, helped teachers adapt to working with others and adjusting to their environment. Interpersonal skills also promoted the development of other skills, such as academic, technical, and professional competencies. Teachers working in multicultural societies needed a broader vision, a positive attitude, and the ability to recognize cultural diversity in the classroom. They needed to be familiar with diverse classrooms and promote harmony among students. As a result, soft skills served as professional support for teachers who understood their students, admired diversity, and recognized the dynamic link between students and their surroundings [17].

### Internationalization of education

The internationalization of education emerged as a response to globalization, though no consensus existed on its interpretation. Knight and de Wit [18] defined internationalization as the integration of international, intercultural, and global dimensions into postsecondary education. This dynamic process operated at multiple levels, influencing policy, curriculum design, and institutional strategies.

While the concept of internationalization in higher education aimed to broaden students’ life experiences through exchanges and international learning, people often used it interchangeably with terms like global education and global citizenship. In primary and secondary education, internationalization was closely associated with the development of global competencies. Extensive research and debates on internationalization primarily focused on higher education, but in the context of school education, it involved transforming schools to adapt them to an international context through transnational cooperation.

Although internationalization was generally considered a positive phenomenon, it could have unintended negative consequences, such as reinforcing stereotypes. Therefore, it was crucial to prioritize quality experiences and initiatives over a purely quantitative approach. The internationalization of higher education not only enhanced business competitiveness but also fostered the development of comparative skills [19, 20]. Exposure to diverse cultures and different ways of thinking enhanced students’ intercultural, critical thinking, and problem-solving abilities, enabling companies to operate globally and adapt to varying business cultures.

Engaging in internationalization activities within educational settings facilitated the formation of multicultural work teams and the acquisition of intercultural communication skills, adaptability, and empathy. Students gained knowledge about other cultures and developed a greater appreciation for diversity, highlighting the importance of their participation in such activities.

Furthermore, internationalization was closely linked to promoting inclusion and diversity in higher education through an understanding of multiculturalism. Practical strategies, such as strengths- and narrative-based approaches, effectively fostered intercultural skills and promoted inclusion in teaching and learning processes.

Finally, the internationalization of higher education opened new possibilities for connections between scientists worldwide who were linked to R&D&i projects addressing common challenges. This connection between scientists beyond borders was promoted by the diplomatic missions of different countries as an instrument of foreign policy to defend national interests and overcome global challenges. This practice became known as Science Diplomacy.

In Latin America, science diplomacy was framed as an “umbrella concept” encompassing policies, activities, and processes that connected science, foreign policy, and international relations [21]. However, effective science diplomacy required specialized training, which remained a challenge in the region. Training researchers in multicultural skills and attitudes served as a pathway to enhancing science diplomacy efforts.

Although the concept was relatively recent, originating from a 2010 report titled *New Frontiers in Science Diplomacy* by the Royal Society and the AAAS, a growing consensus highlighted the importance of coordination between the spheres of science, technology, innovation, and international relations to jointly address global challenges such as climate change, biodiversity loss, the transition to clean energy, and health crises like the Covid-19 pandemic. However, the practice of science diplomacy required specific skills in diplomats and scientists, which posed a challenge in Latin America due to the absence of formal training programs in the subject.

Particularly in the case of researchers, training in multicultural skills and attitudes represented a way to approach science diplomacy. Multicultural skills and science diplomacy were linked and had a positive relationship necessary to achieve connections between scientific communities worldwide around global challenges.

### Multicultural attitudes in teachers

Multicultural education encouraged respect, inclusion, and appreciation of cultural diversity in academic settings [22]. Educators today faced various challenges and opportunities within the evolving educational landscape. To effectively respond to these changes and meet the diverse needs of students, teachers needed not only technical knowledge but also flexibility, adaptability, and soft skills [23]. Higher education institutions strived for relevance by preparing students for the demands of the labor market while fostering global perspectives, inclusivity, and civic awareness.

Accessibility to higher education needed to be ensured for students from all socioeconomic and cultural backgrounds, promoting diversity, intercultural dialogue, and acknowledging the value of different experiences [24]. Multicultural education surpassed mere interaction and aimed to establish collaborative relationships within genuinely multicultural environments [25]. It promoted social equity, educational equality, and social justice, thereby benefiting students’ moral and physical development [22].

Teachers played a crucial role in managing instruction and interaction with culturally and linguistically diverse students. Key elements for effective teaching in diverse classrooms included teachers’ self-awareness of their ethnic and linguistic identity, setting high expectations, and incorporating diverse perspectives into the curriculum [22]. Teachers needed training in global citizenship and multiculturalism, and educational policies needed to reinforce these values [16].

Soft skills such as effective communication, problem-solving, and adaptability were highly sought after by employers, yet schools and universities often lacked adequate teaching of these skills [26]. Teachers needed to recognize the importance of developing soft skills in students for their employability and personal growth.

Understanding the motivational factors in global education was essential for effective teaching. Personal connections to global issues, perceived relevance, curiosity, a sense of responsibility, and the practical utility of knowledge were key motivating factors [16]. Active and participatory teaching approaches, authentic learning experiences, and critical dialogue enhanced student learning and motivation.

Teachers’ multicultural attitudes, their ability to adopt different perspectives, and culturally sensitive teaching were interconnected. When teachers developed positive attitudes toward the culturally diverse world and the ability to understand different visions, they generated more culturally sensitive teaching in their students [27]. Therefore, educational institutions needed to ensure teacher training that developed these skills, leading to the improvement of multicultural knowledge and tolerance.

When teachers had a greater multicultural attitude, they developed the ability to adopt different perspectives and understandings of the world, which helped them improve their teaching based on the importance of the cultural context. Including these aspects within curricula and educational policies was important for the development of interpersonal skills and inclusive learning environments that embraced global cultural diversity [16].

### Multicultural attitudes in students

Multicultural education positively influenced students’ perceptions of their school environment, fostered appreciation for cultural diversity, and enhanced social and emotional skills. However, disparities in access to multicultural education could exacerbate inequalities, particularly in resource-limited educational settings [28]. Students who lacked exposure to diverse cultural perspectives might develop limited worldviews, reinforcing pre-existing biases and inhibiting cross-cultural understanding [25].

The integration of information technologies helped bridge the digital divide and facilitated intercultural interactions [25]. Universities played a crucial role in expanding students’ global and cultural knowledge, equipping them with interpersonal and critical thinking skills essential for navigating multicultural environments. Multicultural education aimed to transcend national boundaries, promoting unity, equity, and an appreciation for cultural differences.

Despite its benefits, several barriers hindered the effective implementation of multicultural education. Language differences, communication challenges, and institutional constraints could limit the ability of students and teachers to engage meaningfully with multicultural curricula. Addressing these challenges required targeted educational policies, professional development for educators, and inclusive pedagogical approaches that fostered global perspectives among students [22].

Ultimately, teachers played a vital role in shaping students’ multicultural attitudes. By fostering culturally inclusive classrooms and integrating diverse perspectives into their teaching, educators prepared students for an increasingly interconnected world. Educational institutions needed to prioritize the development of multicultural competencies to ensure that students and teachers alike could thrive in diverse and dynamic learning environments [29].

### Relationship between soft skills and multiculturalism

In the digital age, with artificial intelligence at our fingertips, strengthening soft and interpersonal skills became increasingly important for professional development. Despite this recognition, many professionals, teachers, and students had not developed these skills [30], making it necessary to explore the factors that promoted soft skills such as communication, collaboration, critical thinking, creativity, and problem-solving [31]. Similarly, it was essential to shift from personality studies and psychological factors to social factors such as social support [32].

Reading skills, text analysis, the use of various sources of information, and technological skills such as working on shared documents and mastering digital platforms were essential for the success of graduates in the 21st century [32, 33, 34]. Learning these types of skills was necessary to interpret social relationships, leadership, creativity, and critical thinking in the local and international labor market [35].

Experiential learning approaches, problem-based learning models, and self-managed learning modules were proposed as effective strategies to enhance skills transfer and bridge the gap between the classroom and the workplace [36]. Although 21st-century skills extended beyond the digital realm, technology played a significant role in their development [37].

The transferability of 21st-century skills from a learning context to real-world applications and their successful implementation depended on various factors [38]. Eyal et al. [39] identified three key factors that influenced the quality of online courses: reducing distance from the teacher, the learning environment, and peers. Problem-solving skills could be optimized through “think aloud” processes that involved metacognitive strategies and emphasized skill transfer [38].

Amzalag and Masry-Herzallah [40] found that the success of skills transfer depended on the cultural dimension of education, which included cooperative education, creative teaching, motivation to teach, teacher empathy, and the successful implementation of a multicultural approach to teaching and learning processes.

Teachers with strong soft skills demonstrated greater adaptability in multicultural classrooms, fostering inclusive learning environments. Previous studies indicated that interpersonal skills, such as empathy and communication, positively impacted multicultural attitudes [17,41,42]. Furthermore, higher education institutions needed to integrate soft skills training into faculty development programs to enhance multicultural competencies.

Soft and interpersonal skills had a positive correlation with students’ employability [12]. Incorporating intercultural education into the training of future managers and business leaders better prepared them to navigate an increasingly diverse and globalized world. By emphasizing communication and promotion strategies for multicultural environments, higher education institutions played a key role in ensuring that these leaders were equipped to thrive in such contexts. In short, intercultural competence and soft skills guaranteed success in multicultural environments and the global labor market [43, 44].

An example of this occurred in transnational corporations, which mostly hired graduates with relevant skills and competencies, underlining the importance of the quality of higher education to meet employment needs [45]. For this reason, higher education institutions needed to encourage training in practical skills and competencies to improve the employability and global competitiveness of their graduates [45].

Understanding these mechanisms that made students more competitive required the adoption of a multidisciplinary approach by teachers so that they could offer students a comprehensive understanding of the functioning of global cultures from different perspectives. Therefore, the soft skills that teachers strengthened in their students needed to promote adaptation to different work cultures, understanding of diverse perspectives, and collaboration within teams. A pedagogical approach that integrated cultural perspectives, scientific diplomacy, gender studies, sustainability, and diversity in education encouraged critical reflection and awareness of social and environmental issues. Similarly, it facilitated intercultural understanding and tolerance.

When interpersonal skills and multicultural attitudes were not encouraged in higher education institutions, barriers arose in learning with culturally diverse students. This situation made them less competitive in some contexts. For this reason, universities needed to emphasize training programs that developed interpersonal skills and promoted multiculturalism in the classroom, improving the quality of teaching and preparing students for a diverse and globalized world. Complementarily, science diplomacy should be proposed from an interdisciplinary approach, as it required not only science and diplomacy but also the development of other skills such as negotiation, decision-making, and working with intercultural teams [46]. Additionally, other types of skills, such as leadership, critical thinking, networking, and assertive communication, were necessary.

Educating in science diplomacy required exploring the knowledge and skills that scientists and diplomats needed to learn from and about each other to work more closely together [46].

In conclusion, this literature review emphasized the importance of developing soft skills and intercultural competence and adopting a multidisciplinary approach to multicultural education in higher education. By highlighting the significance of practical skills, intercultural understanding, and global perspectives, it provided valuable insights for educators, employers, and students seeking to enhance employability and thrive in multicultural environments.

## Method, model, and identification strategy

This research evaluated the effects of multicultural ideology, soft skills, national pride and identity, and intergroup contact using an educational production function. The conceptual framework of this function established a linear relationship between the inputs used in the educational process and the school output at the teaching level [47]. The study developed and estimated this objective through correlation analysis and the method of ordinary least squares (OLS). It aimed to verify how multicultural ideology, national pride and identity, and intergroup contact, while controlling for sociodemographic variables, influenced the multicultural attitudes of teachers.

The identification strategy is the following:


$$\begin{array}{l} MulticulturalAttitudesi\\ \;=\beta 0+\beta 1SoftSkillsi+\beta 2Multiculturalideo\log yi+\beta 3ethnici\\ +\beta 4Nationalpridei+\beta 5Agei+\beta 6Genderi+\beta 7outsidecountryi+\mu i \end{array}$$ (1)

According to the research question, the dependent variable is an indicator of multicultural attitude, *Multicultural Attitudesi*. It will be evaluated if an indicator of soft skills (*Soft Skillsi*), of multicultural ideology (*Multicultural ideologyi*), the knowledge of friends and peers in other ethnic groups (*ethnici*), and national pride (*National pridei*), affect the dependent variable. Variables related to age (*Agei*), gender (*Genderi*), and Participation in activities outside the country (*outside countryi*) are included in this model.

The information comes from the collection of primary information by the teachers who work in a recognized high-quality Colombian university, based on the following instruments:

1. The Teachers’ Multicultural Attitude Survey (TMAS: Ponterotto et al. [48]).
2. The Multicultural Ideology Scale (MIS: Berry, Kalin, & Taylor [49]; Berry & Kalin [50]).
3. Perception list of the use of soft skills in teachers according to the framework of the World Economic Forum and UNESCO
4. The National Identity Survey (International Social Survey Programme [51].

The Teachers’ Multicultural Attitude Survey (TMAS) is a tool designed to assess the multicultural attitudes of teachers. It was developed by Ponterotto et al. in 1998 [48]. The TMAS has 30 items to measure teachers’ perceived attitudes toward cultural diversity, including attitudes toward minority students, ethnic identity, and cultural differences. This survey also contains aspects of multicultural sensitivity, Multicultural Competence, and Multicultural Attitudes/Beliefs.

The Multicultural Ideology Scale (MIS) measures individuals’ attitudes toward multiculturalism and diversity. The instrument developed by Berry et al. [49] has 20 elements that highlight the recognition of cultural diversity, respect for cultural diversity, and cultural pluralism. It is useful in research to examine attitudes toward multiculturalism and diversity in a variety of contexts, including education, business, and healthcare.

Soft skills are the transversal capacities that entrepreneurs will demand in the context of the new industrial revolution. In this research, we stick to a set of activities planned by the World Economic Forum and UNESCO, listed in:

1. Analytical thinking and innovation.
2. Active learning and learning strategies.
3. Resolution of complex problems.
4. Critical thinking and analysis.
5. Resilience, stress tolerance, and flexibility.
6. Creativity, originality, and initiative.
7. Leadership and social influence.
8. Reasoning, problem-solving, and ideation.
9. Emotional intelligence.
10. Technology design and programming.
11. Assertive communication
12. Negotiation
13. Interpersonal and intercultural skills
14. Knowledge of global issues

The soft skills variable in the model collects the simple average of the qualifications that the teacher recognizes as its strengths.

The National Identity Survey consisted of a set of questions that were designed to assess people’s national identity, including questions about their sense of belonging to their country, their pride in their country, their attitudes towards other countries, and their views on immigration and globalization. The survey also asked questions about political attitudes, such as trust in government and support for democracy.

Finally, Intergroup contact was measured through two items.

a. The first item addressed experiences of being in multicultural classrooms (Have you had classmates of other ethnicities in all your years of study (primary, secondary, university education) (based on studies in mono- and multicultural classrooms, cf. Dutton et al. [52]).
b. The second item addressed intergroup friendship (How many close friends of the same and a different ethnicity than yours do you have?) (cf. Berry [53]).

A series of questions related to international cooperation and scientific diplomacy are included in the format, in addition to specific controls for the identification of the respondent.

The research adapted this form to the context of a high-quality Colombian university accredited by the Colombian Ministry of Education in 2021. This institution featured multicampus cultural diversity and was characterized by its inclusive, diverse, and transformative approach to student development. The survey targeted the teaching population of the Faculty of Administrative, Economic, and Financial Sciences, which consisted of 270 teachers delivering virtual and face-to-face instruction across 23 undergraduate and master’s programs. The random and probabilistic sample included 199 teachers, corresponding to a confidence level of 99% and a permissible sampling error of 4.7%.

The processing of personal data complied with Colombian Law 1581 of 2012, which regulates the protection of personal data. The research guaranteed confidentiality, protection of identity, and the good name of participants, adhering to principles of security and confidentiality. The data obtained through surveys were anonymized, ensuring that the identity of respondents could not be traced. This approach safeguarded their privacy and rights in accordance with current regulations.

## Results

Table 1 presents the descriptive statistics of the main variables obtained from the survey of 199 professors at an accredited, high-quality Colombian multi-campus university. The multicultural attitude composite indicator averaged 3.5, indicating a notable level of agreement with statements related to multicultural attitudes. The table also shows that 57.3% of the surveyed teachers were men, with an average age of 44 years (ranging from 27 to 69 years). The average score for transversal skills was high (4.3), but a notable below-average pattern emerged in the area of technology design and programming. Finally, the scores for multicultural ideology and national pride were below 3.6 out of 5.

**Table 1:** Descriptive statistics.

Variable	Obs	Mean	Std. Dev.	Min	Max
Multicultural Attitudes index	198	3.517	.607	1.4	5
Gender	199	.573	.496	0	1
Age	199	44.05	7.68	27	69
Participation in activities outside the country	199	.437	.497	0	1
Soft Skills index	199	4.294	.608	1	5
Multicultural Ideology index	197	3.471	.581	1.4	5
ethnic groups	195	.728	.446	0	1
National pride index	193	3.579	.616	1	5

Source: Multiculturalism Survey, 2023

Table 2 reports the correlations between the variables of interest. The results revealed positive and significant correlations between soft skills and the Multicultural Ideology Index. Similarly, significant but inverse relationships emerged between multicultural attitudes and knowledge of friends and peers from other ethnic groups. Additionally, a 40% positive and significant correlation appeared between soft skills and the Multicultural Ideology Index, while the Multicultural Ideology Index correlated negatively with knowledge of friends and peers from other ethnic groups and positively with the national pride index.

**Table 2:** Pairwise Correlation table.

Variables	(1)	(2)	(3)	(4)	(5)	(6)	(7)	(8)
(1) Multicultural Attitudes	1.000							
(2) Gender	0.011	1.000						
(3) Age	−0.018	0.098	1.000					
(4) Participation in activities outside the country	0.058	−0.058	−0.006	1.000				
(5) Soft skills	0.419*	−0.088	0.089	0.071	1.000			
(6) Multicultural Ideology index	0.517*	−0.044	−0.042	0.008	0.399*	1.000		
(7) ethnic groups	−0.173*	0.010	0.057	0.119	0.033	−0.246*	1.000	
(8) National pride index	0.139	−0.004	0.059	−0.051	−0.013	0.183*	0.057	1.000

***p<0.01,

**p<0.05,

*p<0.1 Source: Multiculturalism Survey, 2023

Attitudes toward multiculturalism in educational contexts are desirable qualities for effective teaching practices and understanding student learning dynamics. The results in Table 3 indicate a positive effect of soft skills, national pride, and multicultural ideology on teachers’ multicultural attitudes, while knowledge of friends and peers from other ethnic groups had a negative effect. The R-squared values of the regressions with robust standard errors (to account for heteroscedasticity) ranged between 33% and 34%, indicating moderate explanatory power. The mean variance inflation factors (VIFs) for the independent variables in the linear regression model ranged between 1.12 and 1.18, suggesting no significant multicollinearity issues. These results align with interpretations found in the literature review.

**Table 3:** Least ordinary squares regression.

	(1)	(2)	(3)	(4)
VARIABLES	Multicultural Attitudes	Multicultural Attitudes	Multicultural Attitudes	Multicultural Attitudes
Soft skills	0.323***	0.326***	0.333***	0.330***
	(0.0899)	(0.0891)	(0.0870)	(0.0857)
Multicultural Ideology index	0.370***	0.368***	0.367***	0.367***
	(0.111)	(0.109)	(0.109)	(0.109)
ethnic groups	−0.145*	−0.144*	−0.146*	−0.154*
	(0.0828)	(0.0838)	(0.0841)	(0.0875)
National pride index	0.0772	0.0794	0.0800	0.0825
	(0.0690)	(0.0670)	(0.0676)	(0.0688)
Age		−0.00233	−0.00308	−0.00303
		(0.00560)	(0.00584)	(0.00585)
Gender			0.103	0.107
			(0.0754)	(0.0738)
Academic Participation in activities outside the country				0.0602
				(0.0724)
Constant	0.663	0.753	0.694	0.677
	(0.456)	(0.463)	(0.460)	(0.457)
Observations	192	192	192	192
R-squared Mean VIF	0.333 1.18	0.334 1.15	0.341 1.13	0.344 1.12

Robust standard errors in parentheses

***p<0.01,

**p<0.05,

*p<0.1

Age did not have a statistically significant effect on teachers’ multicultural attitudes. However, the negative coefficient suggested that as teachers aged, they might become less open to beliefs and attitudes toward a globalized world. Alternatively, older teachers might find it more challenging to understand the unique needs of students from different cultural backgrounds. This could lead to ineffective teaching strategies and a negative learning environment. A hostile learning environment could harm students from diverse backgrounds, potentially resulting in lower retention rates, reduced academic performance, and dissatisfaction with the higher education institution.

An important finding of this research relates to social skills, such as empathy, communication, and cultural competence. Teachers with strong social skills demonstrated a better ability to understand the unique needs of students from diverse backgrounds. This enabled them to create welcoming and inclusive learning environments for all students and interact more effectively with those from different cultural backgrounds.

Multicultural ideology had a significant and positive effect on teachers’ multicultural attitudes in contemporary societies and higher education institutions. Teachers who recognized the values and beliefs supporting the existence of diverse cultures within a society were better prepared to interact with students and collaborators from diverse backgrounds [54]. These teachers exhibited inherent respect for diversity, inclusion, and equality, which they transmitted in their classrooms. Teachers with stronger multicultural ideologies were more likely to understand cultural diversity and embrace inclusive and welcoming learning practices. These practices fostered an understanding and appreciation of the needs of students from different cultural backgrounds, contributing to their academic success.

Attachment to similar ethnic groups limited the understanding of other cultures and hindered efforts to promote diversity and inclusion. In this study, this variable negatively affected teachers’ multicultural attitudes. These findings align with studies such as Lee and Chen [55], which found that friendship ties between teachers of the same ethnic group could result in a lack of understanding and appreciation of other cultures, undermining efforts to promote multicultural education. Similarly, Sleeter [56] observed that such ties could lead to a lack of diversity in teaching and research, further hindering multicultural education initiatives.

Finally, although national pride was not statistically significant in this study, it has the potential to enhance cultural competence and understanding by promoting a sense of belonging and appreciation for diverse cultures. In higher education, national pride could play a positive role in fostering multicultural attitudes among teachers by improving cultural competence and understanding.

## Discussion

The analysis of the results in the Colombian context allowed us to draw several conclusions about the significant relationships between variables related to multicultural attitudes.

Beginning with the correlations, the significant and positive relationships between multicultural attitudes, interpersonal skills, and the multicultural ideology index indicated that these factors aligned in promoting inclusive teaching practices among teachers. However, the inverse relationships between multicultural attitudes and knowledge of friends and peers from other ethnic groups suggested potential challenges or biases in intergroup interactions.

The positive effects of social skills, national pride, and multicultural ideology underscored their importance in fostering multicultural attitudes. In contrast, the negative effect of knowledge of friends and peers from other ethnic groups highlighted the potential influence of echo chambers and homophily. The inclusion of multiple interpretations from the literature acknowledged the complexity of these relationships. These findings led us to propose timely recommendations for teacher training and professional development. Specifically, we emphasized the need for programs that enhance effective interaction with diverse student populations and strengthen interpersonal skills to foster multicultural attitudes among teachers, thereby creating welcoming and inclusive learning environments [57].

The positive relationship between multicultural ideology and multicultural attitudes reflected the importance of diversity and inclusion. Therefore, higher education institutions needed to ensure the development and promotion of activities rooted in multicultural ideology. These activities should include responses to diversity and inclusion, open dialogue, critical reflection, and professional development for teachers. On the other hand, the negative relationship between attachment to similar ethnic groups and multicultural attitudes called for programs and strategies that promoted intercultural interactions and tolerance of multiculturalism in a respectful manner. Recognizing cultural diversity as a key interpersonal skill in the 21st century, along with fostering understanding, tolerance, and intercultural collaboration, became an instructional priority for teachers. These efforts aimed to develop a more inclusive mindset among students.

In summary, the results encouraged higher education institutions to prioritize the creation of more welcoming and equitable learning environments that embraced cultural diversity. These spaces provided opportunities for students and teachers to share their cultural backgrounds and integrate inclusive teaching practices into the curriculum.

## Conclusions

This research found that teachers’ multicultural ideology, interpersonal skills, national pride and identity, and intergroup contact, when controlling for other variables, were significantly related to their multicultural attitudes. The results demonstrated a positive effect of interpersonal skills, national pride, and multicultural ideology on teachers’ multicultural attitudes, while knowledge of friends and classmates from other ethnic groups had a negative effect.

The findings highlighted the need for differentiated policies. For instance, reactions toward multiculturalism in educational settings continued to be influenced by determinants such as cultural diversity, personal experiences, intercultural teaching, social and cultural influences, and educational policies. Higher education institutions must consider these factors when designing programs and activities that promote multiculturalism in classrooms. Teachers, through their actions and the experiences they provide to students, must foster positive attitudes toward multiculturalism, promoting intercultural education and cultural awareness.

The results revealed a negative impact of knowledge of friends and colleagues from similar ethnic groups on multicultural teaching attitudes. For this reason, training and support for teachers and staff became essential to increase their awareness and understanding of different cultures and perspectives. With this foundation, the next step would involve implementing multicultural strategies in curricula and pedagogical activities.

This research underscored the significant positive impact of multicultural ideology on multicultural attitudes in higher education. A welcoming and inclusive learning environment led to better student retention rates, improved academic performance, and greater overall student satisfaction. Additionally, the study highlighted an aspect rarely explored in the literature: the positive and significant impact of interpersonal skills on multicultural attitudes in higher education. Faculty members with strong social skills could create a positive and welcoming learning environment for all students, resulting in higher retention rates, improved academic performance, and greater student satisfaction.

The findings reflected the influence of soft skills and the tolerant global context acquired throughout a teacher’s life on multicultural attitudes. However, understanding the mechanisms that promote these good practices remained crucial. This research proposed, as a monitoring and impact policy, the development of science diplomacy and internationalization strategies to strengthen the skills teachers acquire in the context of the new industrial revolution. Future research should address the direct relationship between science diplomacy, soft skills, and multicultural attitudes.

Finally, the findings of this research provided practical recommendations for higher education institutions seeking to enhance teacher training in interpersonal skills, multicultural awareness, and inclusive educational policies. For example, implementing structured training programs that focus on multicultural sensitivity and the development of interpersonal competencies could enable educators to better understand and value cultural diversity, fostering more inclusive learning environments [14]. Similarly, activities emphasizing multicultural values were crucial in promoting positive attitudes toward diversity within teacher training programs. Furthermore, internationalization and science diplomacy served as complementary tools that could reinforce these skills and prepare educators for a globally interconnected educational context. This study recommended that future research further investigate the relationship between science diplomacy, soft skills, and multicultural attitudes, with an emphasis on identifying effective strategies for advancing multicultural education.

In conclusion, this research underscored the importance of soft skills, multicultural ideology, and national pride in shaping teachers’ multicultural attitudes. The findings highlighted the need for faculty development programs that integrate soft skills training and intercultural education. Additionally, promoting inclusive policies and international collaboration in higher education could further support multicultural competence among educators. Future studies should explore science diplomacy and internationalization as complementary strategies for fostering multicultural attitudes in academic institutions.

## Study limitations and future research

Despite its contributions, this study has limitations that should be addressed in future research. First, the study relies on cross-sectional survey data, limiting the ability to infer causality. Future studies should adopt longitudinal designs to examine changes in multicultural attitudes over time. Second, qualitative approaches, such as in-depth interviews and focus groups, could provide deeper insights into the contextual factors influencing teachers’ multicultural perceptions. Finally, comparative studies across different educational institutions and cultural settings would enhance the generalizability of findings.

## Supporting information

S1 File Multiculturalism Survey. (DTA)

S1 Text Multiculturalism Survey. (TXT)

S1 Data Multiculturalism Survey. (XLSX)

S2 Text Multi.do. Supporting information contains a Stata do-file with the programming code used to generate the tables presented in the manuscript [58].
(DO)
